# Younger adult women who had a stroke or at high stroke risk: Exploration of their experiences and needs

**DOI:** 10.1016/j.qrmh.2025.100034

**Published:** 2025-12-16

**Authors:** Sarah Ibrahim, Emine Kocabas, Lindsey Zhang, Angela Verven, Syeda Hashmi, Sharon Ng, Troy Francis, Aleksandra Stanimirovic, Judith Coulson, Jasper R. Senff, Jonathan Rosand, Sanjula D. Singh, Valeria E. Rac, Aleksandra Pikula

**Affiliations:** a Program for Health System and Technology Evaluation, Toronto General Hospital Research Institute, Toronto, Ontario, Canada; b CANadian consortium of clinical trial TRAINing (CANTRAIN), Research Institute of the McGill University Health Centre (RI-MUHC), Canada; c Institute of Health Policy, Management and Evaluation (IHPME), Dalla Lana School of Public Health, University of Toronto, Canada; d Centre for Advancing Collaborative Healthcare & Education, University of Toronto, Toronto, Ontario, Canada; e Jay and Sari Sonshine Centre for Stroke Prevention & Cerebrovascular Brain Health, Toronto Western Hospital, University Health Network, Toronto, Ontario, Canada; f Faculty of Medicine, University of Ottawa, Ottawa, Canada; g Faculty of Medicine, University of Toronto, Toronto, Canada; h Henry and Allison McCance Center for Brain Health, Massachusetts General Hospital, Boston, MA, United States; i Department of Neurology, Massachusetts General Hospital, Boston, MA, United States; j Broad Institute of MIT and Harvard, Cambridge, MA, United States; k Center for Genomic Medicine, Massachusetts General Hospital, Boston, MA, United States; l Harvard Chan School of Public Health, Boston, MA, United States; m Lawrence Bloomberg Faculty of Nursing, University of Toronto, Canada; n Department of Neurology and Neurosurgery, Brain Center Rudolf Magnus, University Medical Center Utrecht, Utrecht, Netherlands; o Ted Rogers Centre for Heart Research at Peter Munk Cardiac Centre, Toronto General Hospital Research Institute (TGHRI), University Health Network (UHN), Toronto, ON, Canada; p Department of Medicine, Division of Neurology, University of Toronto, Toronto, Ontario, Canada; q Department of Neurology, Toronto Western Hospital, Toronto, ON, Canada; r Krembil Brain Institute, University Health Network, Toronto, Ontario, Canada

**Keywords:** Stroke, Younger adult women, Experience, Qualitative research

## Abstract

**Background:**

Worldwide, women, particularly younger and middle-aged (≤65 years), are disproportionately affected by stroke. Although the adoption of healthy lifestyle habits is integral for stroke risk factor modification, little is known about younger adult women who had a stroke or are at high risk of stroke—their lifestyle-related knowledge, behaviors, associated and influencing facilitators, and barriers to support brain health, which this study aimed to address.

**Methods:**

A qualitative interpretivist design was employed that was part of a larger quality improvement mixed-methods study. Data was collected through virtual, semi-structured focus groups. Inductive thematic analysis was performed and analyzed using the intersectionality framework.

**Results:**

A total of 11 women comprised the study sample who were of high stroke risk or had a stroke (mean age 53 years, 54.5 % stroke). A total of six themes emerged from the analysis: (1) lifestyle habits supporting holistic post-stroke recovery, (2) parenting—not being the same mother as before, (3) professional expectations—having to leave career behind and pressures to return, (4) societal expectations of women and permission to self-care, (5) psychological safety and comfort from women-centered interventions, and (6) experience in the healthcare system—not feeling seen, heard, or considered as a woman.

**Conclusions:**

Study findings have implications on three levels: 1) *micro*, through the development of intensive, long-term educational, behavioral, peer-led, group-based and theory-informed interventions that focus on holistic and incremental lifestyle changes and involve family and social support; 2) *meso*, through the use of practical tools in clinical practice, integration of motivational interviewing and health coaching, and services for children in health care and school systems, and 3) *macro*, through the incorporation of case management and psychosocial support in the current model of stroke care.

## Introduction

Globally, women are disproportionately affected by stroke, as they account for 56 % (in 2019) of all people who experience a stroke (Feigin et al., 2022). This is coupled with an overall higher life-time stroke risk and increasing stroke burden among younger adults (Feigin et al., 2014) and particularly younger adult women (Rexrode et al., 2022, Vyas et al., 2021). Similarly, in Canada, 62,000 strokes occur annually with over 30,200 among women (Heart and Stroke Foundation of Canada, 2018). Women also experience worse outcomes after stroke with respect to mortality and overall disability (quality of life, poststroke depression, and activity limitations) (Rexrode et al., 2022). Yet, women’s brain health has historically been understudied (Beery and Zucker, 2011, Caldwell and Isenberg, 2023), and women are underrepresented in stroke research (Heart and Stroke Foundation of Canada, 2018). The current sex biases and gender inequities in stroke research, which has been referred to as the “brain health gap” (Berkhout et al., 2024a), has the potential to negatively impact the provision of optimal brain health and care for women, compromising the scientific potential and promise of discovery to inform the development of novel innovations, interventions, and efficacious initiatives for women (Galea et al., 2020, Taylor et al., 2019). This was illustrated in a perspective piece presenting the misdiagnosis of a stroke in a younger adult woman in a Canadian healthcare system (Berkhout et al., 2024a).

The distribution of stroke risk among women is complex and multi-faceted compared to men (Heart and Stroke Foundation of Canada, 2018, Yoon and Bushnell, 2023). For instance, there are sex-related differences such as history of adverse pregnancy outcomes (e.g., gestational hypertension), early/late menarche, and oral contraceptives with estrogen that increase stroke risk for women (Rexrode et al., 2022). This is further coupled with modifiable and non-modifiable (e.g., genetics, age, and sex) risk factors. Modifiable risk factors (MRFs) such as hypertension, poor diet, low physical activity, and high cholesterol account for 90 % of the global stroke burden (Bukhari et al., 2023, Feigin et al., 2016, Pikula et al., 2024). A large body of literature and guidelines such as those of the Heart and Stroke Foundation of Canada (Heart and Stroke Foundation of Canada, 2021) and the American Heart Association (Kleindorfer et al., 2021, on behalf of the American Heart Association, 2022) support the importance of healthy lifestyle habits to target MRFs to improve overall health, wellbeing, and quality of life (Rippe, 2018) and to reduce stroke risk and recurrence (Bondonno et al., 2021, Pikula et al., 2024, Rees et al., 2019).

The adoption of healthy lifestyle habits is influenced by a myriad of factors—individual, community, social, environmental, and economic (Baruth et al., 2014, Ndejjo et al., 2022). The influence of such factors was illustrated in a cross-sectional study conducted by this research team that explored the lifestyle-related knowledge, habits, and associated facilitators and barriers to the adoption of healthy lifestyle habits among younger adult stroke and neurological patients (Ibrahim et al., 2025). Findings from the study yielded variability with lifestyle-related knowledge such as poor knowledge of nutrition and physical activity recommendations and the adoption of healthy lifestyle habits. Notably, emotions (e.g., anxiety, sadness), time constraints, energy levels, and fear were barriers experienced with the adoption of healthy lifestyle habits. Similarly, in a narrative review that provided an overview of factors that influence the promotion of physical activity (PA) among patients and healthcare providers (HCPs) in primary care, the barriers centered around three levels: *individual* (pre-existing health conditions, knowledge of PA-related benefits, time and capacity), *societal* (cultural norms and social support), and *environmental* (e.g., weather, availability of facilities and resources) (Leese et al., 2023).

There is a scarcity of research exploring facilitators and barriers for the adoption of healthy lifestyle habits among younger adult patients who had a stroke, particularly women. Such understanding and exploration are imperative to inform the development of meaningful, applicable, and effective educational, behavioral, and lifestyle-related interventions that meaningfully account for such influencing factors. This, in turn, has the potential to support primary and secondary stroke prevention among this unique population and to propel the paradigm shift of brain health and care for women. The aim of this study was threefold: 1) to explore current lifestyle-related knowledge and practices, 2) to elucidate facilitators and barriers with risk factor management in primary and/or secondary stroke prevention, and 3) to unpack the support needs for brain health and stroke-related interventions among younger adult women who had a stroke or are at high risk of stroke (e.g., previous transient I=ischemic attack [TIA], vascular malformation, or intracranial aneurysm [IA]).

## Methods

### Study design

A qualitative interpretivist design was employed and guided by the ontological position that health-related habits, behaviors, and experiences are socially constructed and shaped by dynamic contextual factors such as physical, economic, political and social environments (Mollborn et al., 2021). This approach assumes that meaning is derived through participants’ lived experiences, which are best understood by actively listening to their insights and perspectives (Patton, 2015). Building on this foundation, this study was framed by the intersectionality theory (Crenshaw, 2013): a framework for understanding how multiple social identities such as gender, age, race, and class interact to shape the unique lived experiences of individuals, particularly in relation to systems of privilege, power, and oppression (Crenshaw, 2013). Rather than understanding and viewing participants’ experiences from a single-axis analysis lens, intersectionality helps explore how the multiple social identities, roles, responsibilities, and experiences of individuals converge within a system that often neglects these complexities. Intersecting identities and factors are not additive, but synergistic, as they produce unique experiences that cannot be understood through the examination of each identity in isolation, supporting a more equity-oriented, inclusive, and responsive approach to women’s health and stroke-related research.

This qualitative interpretivist design was part of a larger quality improvement, mixed methods study (N = 104) that explored lifestyle-related knowledge, behaviors, influencing facilitators, and behaviors regarding healthy lifestyle adoption among younger adult patients who had a stroke and/or people of high risk of stroke. The Standards for Reporting Qualitative Research checklist was used to guide the reporting of the study (O’Brien et al., 2014). Institutional Quality Improvement Review Committee approval (QIRC#23–0518) was obtained for the study.

### Sample, recruitment and setting

The population of interest as part of the larger study was younger adults who at the time of the study were (1) of working age (less than 65 years of age), (2) had a stroke (hemorrhagic or ischemic) more than 90 days prior to recruitment or of high stroke-risk (no stroke, but a diagnosis of TIA, unruptured aneurysm, or vascular malformation), (3) attending the stroke prevention or neurovascular clinics at the academic learning healthcare system focused upon in this study, and (4) able to communicate in English. Younger adults were excluded if, at the time of the study, they had advanced cognitive impairment that would preclude them from providing informed consent and/or who had a trauma-related brain injury (e.g., subarachnoid hemorrhage).

The study was conducted at a stroke center in Canada. Those who participated in the quantitative phase of the larger mixed methods study were invited to take part in the qualitative phase. A convenience sampling method was used. Among the participants in the quantitative phase (N = 104), 23 expressed an interest in the qualitative phase. Participants were contacted by the coordinator to explain the study details and answer any questions and/or concerns. For those who were interested, verbal consent was obtained. Several patients (n = 12) did not respond to the coordinator following three contact attempts. The total study sample was determined based on data saturation, i.e., the emergence of no new themes or data (Saunders et al., 2018).

### Data collection and analysis

Data were collected through focus groups using a semi-structured interview guide (Table 1). Participants were interviewed once by the research team. The focus groups were conducted virtually to reduce associated costs with traveling to the center, coupled with increasing convenience for participants and extending recruitment to reach women residing in different geographic locations (Oliffe et al., 2021). The focus groups were 60–75 min in duration and audio recorded. Identifiable information was removed from the transcripts with an identification number assigned to each participant. A $10 gift card was offered to participants.

**Table 1:** Sample of semi-structured interview questions.

1. What is your current knowledge of a healthy lifestyle? 2. What are your current lifestyle habits and goals? 3. As an individual who identifies as a woman who had a stroke or of high stroke risk, what are your thoughts on the association of the roles and responsibilities that influence the adoption or engagement in a healthy lifestyle? 4. As an individual who identifies as a woman who had a stroke or of high stroke risk, what are your thoughts on the influence of emotions on the adoption of healthy habits? 5. As an individual who identifies as a woman who had a stroke or of high stroke risk, what is your perspective of the influence of responsibilities (specifically professional and personal such as family) on the adoption of healthy habits? How do your current circumstances (e.g., job, income, support) influence your responsibilities and habits? 6. What are your thoughts of unique facilitators to you as an individual who identifies as a woman who had a stroke or of high stroke risk with engaging in a healthy lifestyle? How do your circumstances influence your engagement in a healthy lifestyle? 7. What are your thoughts of unique barriers or challenges to you as an individual who identifies as a woman who had a stroke or of high stroke risk with engaging in a healthy lifestyle? How does your identity, role and responsibilities influence your thoughts on the barriers? 8. Have you participated in any women-focused lifestyle program or interventions and what was your experience like? 9. What suggestions/recommendations do you have for developing or adopting lifestyle intervention or programs for women who had a stroke or of high stroke risk?

The interviewers did not have contact with participants prior to the study. The first and second authors reviewed and coded the transcripts independently. The first author is an assistant professor, scientific associate, and registered nurse with experience in mixed methods and qualitative research; she assumes a pragmatic worldview and identifies as a woman. The second author is a research analyst with qualitative research experience, assumes an advocacy/participatory worldview, and identifies as a woman. Concurrent data collection and analysis were performed to support data saturation (Ravindran, 2019). Inductive thematic analysis was conducted using the six phases outlined by Braun and Clarke, (2006). Validity and reliability of this study was maintained through reflexivity, interview and stepwise techniques, peer-debriefing, code-to-code procedures, investigator triangulation, and thick description (Anney, 2014). The first and second authors discussed the coding and analysis through routinely scheduled meetings and resolved discrepancies through consensus.

## Results

### Participant characteristics

Four focus groups were conducted comprising of three to four women, with a final sample size of 11 participants. All participants were women with an average age of 53 years (range 30–62). Most participants reported being white and Canadian born, with English as their first language. Participants also reported varying levels of child responsibility and most (n = 8) being married. Further, participants’ employment status was mixed, with some (n = 4) being on leave of absence and more than half having an average income of $100,000-$200,000 CAD. Half (54.5 %) of the participants experienced a stroke (Table 2), with others being of high-stroke risk (e.g., diagnosis of TIA, unruptured aneurysm, or vascular malformation).

**Table 2:** Demographic and clinical characteristics.

	**N = 11**
**Age**	
Mean (SD)	53.00 (9.19)
Range	30–62
**Marital Status**	
Missing	1
Single	1
Married/Cohabitating	8
Other	1
**Level of Education**	
Missing	1
Completed High School	2
Completed college/university	4
Post-graduate Degree	4
**Child Responsibility**	
Missing	1
No responsibility	4
Minimal responsibility	1
Moderate responsibility	2
Full responsibility	3
**Canadian or Foreign Born**	
Missing	1
Canadian-born	7
Foreign-born	3
**English Language**	
Missing	1
English first language	9
English second language	1
**Ethnicity**	
Missing	1
White	7
Black	1
East Asian	1
West Indian	1
**Employment Status**	
Missing	1
Manual paid work	1
Student	1
Unpaid volunteer	1
Unemployed, looking for work	1
On leave of absence	4
On disability	1
Not working due to illness/injury	1(10 %)
**Average Income**	
Missing	1
< $15,000	1
$50,000-$99,999	3
$100,000-$200,000	6
**Diagnosed with a Stroke**	
No	5
Yes	6

### Themes and subthemes

**Fig. 1:**
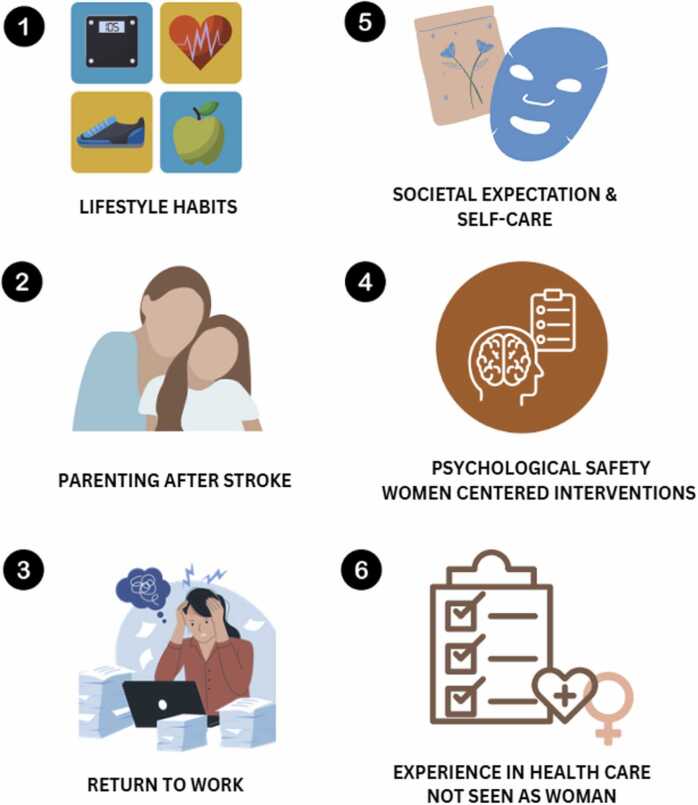
Overview of themes.

Six themes emerged from the data analysis (Fig. 1).

***Theme 1: Lifestyle Habits Supporting Holistic Post-Stroke Recovery***

The nexus of the first theme centered around facilitators and barriers to the adoption of healthy lifestyle habits. Three subthemes comprised this theme: wanting to be healthy for one’s family, not only to live, but to live healthily, and fear and anxiety.

**Subtheme 1a: Wanting to be Healthy for One’s Family.** Participants expressed a strong internal desire to recover and maintain a healthy lifestyle for their families, particularly children and partners. These motivations are deeply rooted in gendered expectations, cultural norms, caregiving roles and responsibilities, and the intersection with their health-related behaviors. The underlying motivators centered around 1) being healthy for their themselves, and family, 2) not being a burden on others and society, and 3) the desire to avoid any further health complications by adopting healthy habits (e.g., eating well, resting, and incorporating physical activity) into their daily routines to support their healing, regardless of where they were in their recovery trajectory. As Participant 1 said:

I don’t want to die. I don’t want to become the burden of other people, of the whole society. I think about my mum, my husband, my son, so this motivates me to go out, to do things. And I don’t want to die at this early age because I think life is beautiful.

This was also echoed by Participant 3:

I would say that my lifestyle habits really do focus on my recovery, I think my biggest habits right now are learning to balance what I feel needs to be done and then, taking care of my mental health and my physical health.

**Subtheme 1b: Not Only to Live, But to Live Healthily.** Participants described a shift in perspective and outlook on life, emphasizing not just survival, but the pursuit of a meaningful, healthy, and fulfilling life. This transformation was influenced by their spiritual practices, community support and culture, and values around health, healing, and wellness. This was expressed by two participants:

Wanting not only to live, but to live healthily. I have to be very careful who I’m keeping company with, so it has to be like-minded people. The social circle, and it’s funny that I never really thought of how important the social circle is, to me, for living a fulfilling life. The social circle is almost everything. It’s the lifeline for people to continue learning. (Participant 2)

Sometimes, before I go to bed, I write it down, what good things happen today and give thanks to God. I never ever write down bad things, so this makes me much happier before I go to bed, and I can sleep very good. (Participant 5)

The different outlooks and coping strategies centered around 1) participants’ perspective towards their recovery, 2) embracing mindfulness, gratitude, and living in the moment, and 3) employing various strategies (e.g., meditation, art, joining a singing group, playing and/or listening to music, and seeking mental health support) to cope with stress and setbacks in recovery. These practices reflect not only individual resilience, but also the influence of broader intersecting social contexts (e.g., community support) and identities regarding how women engage with health-promoting behaviors to support their recovery.

**Subtheme 1c: Fear and Anxiety about Recurrence.** Fear of having another stroke emerged as a significant emotional barrier for participants. Participants described having heightened levels of anxiety and hypervigilance when they would experience symptoms resembling those they had during their stroke (e.g., headache, weakness, and fatigue) or neurological incident. The uncertainty of the cause, severity, and implications of such symptoms created significant emotional distress and disrupted their sense of safety in their recovery. This was captured by two participants:

What can change things is if I get a little headache, which I know is a symptom for me, I go immediately from 0 to 120. It’s like I’m on high alert. I’m like: Is it this kind of headache? Is it bad? Am I close enough to the people that can help me at the hospital? Can I get there quickly, and I can get the help that I need quickly? (Participant 2)

I like to imagine myself driving in a car and something that had happened to me that was my health challenges are receding in the rear-view mirror. With each passing day, there is more distance between me and those events. But I always think about the fact that when will the next event happen?[…] I am trying to both acknowledge those fears while not letting those fears dictate my future. That can just end up paralyzing me. I don’t mean physically. I mean mentally and psychologically. (Participant 7)

These fears may not have been experienced in isolation. Rather, they may have been intensified by the intersection of gendered expectations in addition to implicit bias and the systemic gaps in post-stroke care for younger adults in general and particularly for women.

**Theme 2: Parenting—Not Being the Same Mother as Before**

The second theme centered around the impact of the stroke and/or neurological incident on the family dynamic and household. This theme is comprised of three subthemes: negative impact of stroke on children, overstimulation with mothering/parenting post-stroke, and children adapting to their mother’s stroke.

**Subtheme 2a: Negative Impact of Stroke on Children.** Participants described the emotional and psychological toll their stroke had on their children. Mothers in the sample said that the trauma of witnessing the stroke and the subsequent hospitalization invoked fear, confusion, uncertainty, and emotional distress for the children. This trauma, understandably, had a negative impact on the mothers’ own healing and recovery processes:

My kids struggled a lot because they were here when it happened. So, they saw the onset and had to, you know. And then, seeing me in the hospital, my youngest definitely didn't do well with that. She didn't want to see me when I was in the hospital, but afterwards, it was going to school and having a lot of emotional issues at school because they didn't want to leave me. They were worried. (Participant 8)

Emotions were thus said to be influenced by the children being younger in age (e.g., 4–9 years) coupled with not understanding what had happened to them, and the thought that strokes were contagious. This, in turn, translated into the children’s fear and reluctance to leave their mother, coupled with experiencing emotional distress and challenges in school:

I didn't even know how to tell my kids what had happened […]. Nobody knew how to tell my kids what had happened. So, they don't understand. I tried to give my son a kiss goodnight, and he's like, “Don't come here. You had a stroke.” Thinking of it’s like COVID or something. Kids don't understand. (Participant 6)

**Subtheme 2b: Overstimulation with Mothering/Parenting Post-Stroke.** Participants described feeling overwhelmed and overstimulated by the demands of parenting during their recovery. The sensory overload from the children’s voices, questions, noise, constant movement, and emotional needs created significant distress and often led to frustration and guilt, for example:

Nobody tells you how overstimulating just one or two little voices can be, and it's just the noise that just buzzes around in my head. Now, I'm much better because it's been almost four years, but for the first two years […]. That was brutal. (Participant 7)

While most women in this study were married and reported having supportive partners, which helped buffer some of these emotions and pressures, the feelings of overstimulation and being overwhelmed with the parental demands may be more pronounced for those without such support. For example, for single mothers or women whose social and structural determinants of health are compromised, the lack of adequate space, time, and/or resources (such as the ability to pay for childcare) may hinder their ability to heal and recover, highlighting the impact of their intersecting identities, as explained by Participant 10:

I'm struggling just being a mother, which I could do before. I'm not saying “No problem,” but I was working mother. And I was doing OK. And now, I don't even want to hear their voices. I know that sounds terrible, but I love them. I love them. And I know that they're caught up in all this too, but it's just hard. And no one tells you that.

Participants also articulated their perceived shortcomings in their involvement with their children’s extra-curricular and daily household activities post-stroke, for instance:

I used to be very involved in the kids' schooling, sports, and activities, and since the stroke, I really have to be selective and with what I attend. So, if I'm going to go to a soccer game, I kind of have to plan my time around that. Like what I'll be doing that day because I know that that'll be very stimulating. I never really noticed the motherhood aspect like this. I'm not the same mom as I was before. (Participant 9)

These perceived shortcomings reflect how identity transformation post-stroke intersect with role strain where women must renegotiate their new sense of self within the constraints of their (dis)abilities, recovery, caregiving, and societal ideals of parenthood.

**Subtheme 2c: Children Adapting to Their Mother’s Stroke.** Participants described how their children adapted their behaviors to support their mother’s recovery, which is aligned with the resources and perception elements of the family stress theory (Hill, 1949). This was captured by Participant 2 who said, “My daughter did notice I can't play Connect Four as well as I used to, and she would let me win. And I feel like that's a pretty big thing and wake up call.” This adaptation illustrates how age, developmental stage, emotional intelligence, and family support intersect to influence recovery environments. As another participant reported:

I have young, young children, so they learned pretty early on in my first year that the yelling and screaming and running around when I'm tired, that I would retreat to my room. They learn quite quickly. My daughter was six when it happened, and they want to tell you everything about everything all at once. So, I finally said to her, “Mommy's brain can only handle two things at once, so you have to pick one thing at a time to tell me.” So now they ask, “Mommy, how many things are in your brain right now?” (Participant 4)

This also highlights the emotional burden children carry to support their mother’s recovery. Additional adjustments that children employed, as described by the participants, included not yelling or running around at home too much, being clear and concise with their communication, and giving mothers space to rest and to play quietly.

**Theme 3: Professional Expectations, Having to Leave Career Behind and Pressures to Return**

The nexus of the third theme centered around multifaceted challenges participants faced in relation to their professional identities. Challenges centered around the necessity of leaving careers to prioritize their recovery and well-being and the societal and institutional pressures to return to work. Such challenges had a negative impact on their self-esteem, self-confidence, mental health, and financial stability. This was expressed by a participant who said, “I don't have the same train of thought. I noticed that I have different cognitive abilities. I had a huge work portfolio with a lot of responsibility and to see where I'm at now is very hard for me” (Participant 1). Another participant reflected on the cognitive and emotional toll of this transition:

I tried going back just temporary jobs […]. So, I thought I could try that because it was something that I remembered. But because I lost a lot of my short-term memory and it was difficult fatigue-wise, big time, I would actually have to go to my car during lunch and kind of rest […].I was just too mentally exhausted. (Participant 11)

These narratives show how structural expectations around productivity and recovery often fail to account for the lived realities of individuals navigating (dis)ability, chronic illness, and/or trauma. The pressure to return to work is not merely personal, but also deeply embedded in societal norms that prioritize economic output over an individual’s health and well-being. Moreover, the intersectional lens reveals how access to flexible work arrangements, health care, and social support is unevenly distributed, often reflecting broader patterns of inequality.

**Theme 4: Societal Expectations of Women and Permission to Self-Care**

The fourth theme centers around the societal expectations placed on women and the internalized struggle to grant themselves permission to engage in self-care during their recovery. These experiences are shaped not only by gender, but also the interplay of other social identities such as race, class, age, and (dis)ability which influence how women navigate their health, well-being, personal (e.g., caregiving and parenting), and professional responsibilities. Participants described how deeply ingrained gender norms and socialization positioned them as primary caregivers, resulting in their maintaining household and familial duties even while managing their own recovery. This was captured by a participant who said

As a mom, when you would be ill, you still had to do the laundry. You still had to clean the house. You still had to drive kids around. You still had to do everything that you did before you got sick, but now you have to do it again. (Participant 7)

This illustrates how unmitigated communion (Helgeson & Fritz, 1998)—the tendency to prioritize the needs of others over one’s own—is not a personal trait, but a socially reinforced behavior, particularly among women. This was affirmed by another participant:

Your first thought is your kids and your family and to say “OK.” I got to go to the gym this morning or I've got to go for a walk this morning, but first you got to start your laundry. You got to do your dishes. You got to clean up after the kids have left for school. Then all of a sudden, you're out of energy just doing that, and, then, well, I guess I'll try to try and do it tomorrow. (Participant 3)

Participants also discussed how gendered expectations and biases are introduced early in life and persist across different life stages, shaping women’s identities and how they perceive their roles and responsibilities. This was described by two participants:

I think women are socialized differently too. There is a different expectation, I think, on women to be caretakers and to keep families running at whatever life stage you're in. And so, that does impact where women typically prioritize themselves. (Participant 4)

It's hard to put yourself first when you have children. And I think the other hard part is being pulled in so many different ways, right? We’re not just taking care of kids. We're taking care of the household. We take care of the bills […]. I feel overwhelmed, thinking about having to balance a career, family life, my health, and well-being. (Participant 11)

The concept of granting permission to oneself to engage in self-care had a polarizing emotional impact on participants. For many, this permission was accompanied by feelings of guilt, fear of judgment, and the belief of being selfish or egoistic:

I find that was a really hard thing for me to do was to basically force myself to take time for myself because I felt so guilty that I wasn't doing the things that I used to be able to do. (Participant 8)

From an intersectionality lens, this emotional complexity reveals the convergence of gender norms and biases—caregiving roles coupled with systemic conditions shaping women's approaches to their recovery. Even among women with supportive partners and systems, the emotional impact and internal tension persist. This demonstrates internal and external pressures to remain emotionally available and self-sacrifice being embedded in societal expectations of femininity and motherhood. Notably, for some participants, particularly those who are single parents and whose social and structural determinants of health were more compromised, the emotional and logistical challenges of self-prioritization were even more amplified, highlighting how recovery is not only personal, but deeply political, and shaped by broader systems of inequality.

Interestingly, few participants, particularly those who did not have children and were of higher socioeconomic status, described a shift in perspective as they recognized and acknowledged the importance of setting boundaries and conserving their energy to support their recovery:

I do energy conservation and pacing, so selfishly, I have to use my energy for the things that will benefit my recovery. So that unfortunately means that my partner has to do, again, I don't want to use the term “things that women typically do” because we definitely have different capabilities, but especially at the beginning. (Participant 7)

**Theme 5: Psychological Safety and Comfort from Women-Centered Interventions**

The fifth theme centered around the importance of having women-centered and women-led interventions to support and improve psychological safety and comfort for women during their recovery. This theme was comprised of two subthemes: a) women specific and peer led interventions/programs and b) understandable education on mental health and family care.

**Subtheme 5a: Women-Specific and Peer-Led Interventions/Programs.** The nucleus of participants’ discussions centered around the importance of women-specific and peer-led interventions, which were described as essential to fostering a sense of safety, comfort, connection, and understanding during their recovery. These preferences reflect gendered experiences shaped by intersecting identities including health status, race, (dis)ability, and roles which influence access to care and support systems. Participants consistently emphasized that women-only environments—particularly those led by peers with similar shared lived experiences—offered a unique form of solidarity and unspoken understanding. As Participant 3 explained:

I think women's-only spaces would be valuable where you have that commonality kind of thing. And I think to be able to, just know that people get it even without you having to say it. And it's not that other people don't. But there's a safety and comfort that comes with that for women.

This sense of psychological safety was particularly important for women whose recovery was complicated by societal expectations, demands, and pressures around caregiving, parenting, partnership, and professional responsibilities.

The healing environment of having a women-led program was described by a participant who had previously participated in a women-only cardiac rehabilitative program: “Rehab, which was run by all women. There was an empathy, and an understanding, and kindness. I just found it to be a really good environment to be in at that time during my recovery” (Participant 6). These reflections and discussions highlight how gender-congruent care environments can mitigate feelings of isolation and enhance well-being, quality of life, and recovery outcomes for women navigating complex health journeys.

While participants expressed immense gratitude and appreciation for medical care received during the acute phase of their stroke or neurological event, they consistently emphasized the transformative role of peer support. Peer-led interventions were seen as powerful sources of hope and connection that would allow them to witness others with similar experiences progressing in their recovery. As Participant 7 put it, *“*It's like being able to make that connection with others through lived experience and to talk about our experiences and how we've overcome certain things [that] would help the person who's just starting to go through that journey.” Another participant echoed this sentiment, emphasizing the emotional impact of seeing someone further along in their recovery:

When you’re first sick, you want to get a diagnosis, and you want to get the right medical professionals as you go on your health journey. You need the physicians less often […]. What you’re really looking for is peer support. Somebody who’s further along in your healthcare journey. It gives you hope that they were where you are now, and with time and hard work, and adjustment to lifestyle, look how far they’ve come! It gives you hope. In the end, that’s the four-letter word at the end of it all. (Participant 9)

**Subtheme 5b: Digestible Education on Mental Health and Family Care.** Many participants emphasized the need for understandable and accessible education and information on mental health and family care. It was recommended to have this information during discharge planning and throughout their journey, using various formats such as online platforms, printed handouts, and toolkits. Participant 7, for instance, said, “Have more information in an understandable way, kind of from the jump. And then, also, sort of like a database of resources that you could potentially access online and having a section for women.” Considering that most of the women in this study had higher levels of education and socioeconomic status, there is the potential risk of assuming these recommendations would equally benefit all women. This highlights the importance of reflecting on the intersecting factors such as varying levels of health literacy, language(s) spoken, access to healthcare, and social and structural determinants of health that influence and shape women’s ability to engage with and benefit from such initiatives. For example, a woman with limited English-speaking ability would not benefit from such a resource, but from one that is translated in their primary language.

Beyond general education, participants also expressed a strong need for mental health and family care-related support and resources. This is due to the emotional and psychological impact experienced by the participants and their respective families, coupled with the lack of mental health support, which continues to serve as a roadblock in recovery:

The first day in the stroke ward, I asked for a psychologist because I knew that it was going to be my challenge, and they're, like, “What do you mean?” I'm, like, “I need to speak to someone. I need help to understand what's going on.” And I pushed for a psychologist as someone that's skilled in this area that can help, as it’s a grieving process. There's so much wrapped into that recovery that I was told I had to go find my own. (Participant 9)

The lack of resources and support available through the public healthcare system resulted in some participants resorting to paying out of pocket for care (e.g., vision-related) and services (e.g., therapy) that were not covered by provincial health insurance. The participants did acknowledge that their ability to seek out and access support was influenced and shaped by their privilege such as having higher education, being fluent in English, and having health literacy, access to transportation, and financial means. The lack of mental health support was further amplified with the lack of guidance on available services beyond the acute care setting, leaving participants to navigate these options on their own while still managing their varying post-stroke sequalae. This was expressed by Participant 11:

I'm going out and searching for the care, and I'm paying out of pocket for the care because it's not covered under OHIP which drives me crazy. Because if you can help in, like, repair the vision and the cognition and those pieces, then, I will likely return to work and to regular everyday life.

The same sentiment was expressed by Participant 5:

I think the part that frustrates me the most is that if I wasn't this type of person to go after these things, then I would still be sitting at home, and no one cares. People care about me, but the healthcare system doesn't care.

While there were frustrations with having to search and pay for out-of-pocket services, participants described the benefits, for example:

I see a therapist regularly. It is amazing if you can find yourself someone you connect with, whether it is covered by OHIP [provincial health insurance plan] or you pay out of pocket. It is one of the most beautiful resources I believe anyone can have in their toolbox. (Participant 6)

Participants also emphasized the need for family-related care education and resources, particularly regarding the adoption of healthy lifestyle habits, what to expect when returning home after a stroke, common challenges they may experience (e.g., fatigue, overstimulation, sensitivity to noise), how to communicate with their child, and arranging child support (e.g., school, community) upon discharge, for instance:

Even some resources to explain to a child what has happened to you and I think just like some resources to be to be able to explain that to them or to give it to us, to be able to explain to them or something. (Participant 9)

These reflections demonstrate how recovery is not an isolated experience and is deeply embedded in caregiving roles and family dynamics disproportionately shouldered by women and compounded by social and structural determinants of health.

**Theme 6. Experience in the Healthcare System: Not Seen, Heard or Considered as a Woman.** The sixth and final theme centered around the challenges encountered with navigating the healthcare system as women. This theme is comprised of three subthemes: misdiagnosis of women in the healthcare system, a huge administrative burden on women, and one size does not fit all.

**Subtheme 6a: Misdiagnosis of Women in the Healthcare System.** Many participants described how their stroke symptoms were not taken seriously for several hours and being repeatedly dismissed for vertigo, hormones or anxiety, and/or misdiagnosed for several months despite being seen by multiple HCPs in several healthcare institutions. The HCPs’ approach appears to reflect a perspective and approach in which they struggle to interpret the presenting symptoms as something serious such as a stroke, particularly in younger adult women due to their intersecting social identities: Some, being White, of higher socioeconomic status, younger in age, and fluent in English may not fit the expected profile of a stroke diagnosis. This approach reflects broader systemic issues rooted in gender bias and intersecting forms of marginalization that influence women’s health and related concerns are perceived and addressed. One participant recounted:

I was misdiagnosed for several hours and wasn't taken seriously, even though I had already had a stroke history. I'm still struggling with that. It was seven hours before they had actually physically intervened. I try not to go down the pathway that [it] is because I'm female. But you do wonder whether or not females are taken seriously. They had initially treated me like I had vertigo or something like that. And, things were very serious. I was losing my hearing. I'd lost my vision. There were a lot of things happening at the time. I couldn't stand up. (Participant 3)

This participant’s reflection captures the emotional toll and trauma of being dismissed and the internal conflict of questioning whether her gender played a role in the delayed response. Some who had the financial means to do so resorted to paying out of pocket for imaging to confirm their stroke:

We know statistically as women we are sometimes not taking as seriously by healthcare providers. I was misdiagnosed for three months despite going to eight different healthcare providers, including emergency department twice. And it was the thought of being hormonal or anxious and not being taken seriously. I actually had to pay for my own MRI to show people I actually had a stroke. (Participant 2)

This case highlights how systemic gender bias interests with economic privilege. While some women in this study were able to pay out-of-pocket for diagnostic imaging, this option is not available to many, particularly to those who are socially and structurally marginalized.

**Subtheme 6b: A Huge Administrative Burden on Women.** Discussions centered around the administrative burden placed on women following a stroke, which emerged as a major source of stress and barrier to their recovery. Notably, participants described the overwhelming responsibility of coordinating healthcare-related paperwork to ensure forms were sent to specialists, insurance companies, and employers while simultaneously managing their own recovery as well as parental and household responsibilities:

I'm the go-between for all the doctors: running forms, sending forms. And that stresses me out because, competently, I can do it most days. But a lot of times, I struggle with dates, and times, and where I'm going from one day to the next. I mess up appointments, and I just feel like there's not a lot of help in that way. What I'm asking for is something that should be standard of care, and it's not being done. So now, I'm having to follow up. And I'm now chasing people so I can get ahead in my recovery because I'm literally waiting for them to fax a document. (Participant 4)

This burden was further compounded by cognitive challenges such as memory loss and fatigue which made navigating appointments and related administrative work more difficult. It is important to acknowledge that the women in this study possessed the language skills, health literacy, and some level of knowledge of navigating the system that enabled them to coordinate and communicate accordingly. The administrative burden was a hinderance on participants’ ability to engage in essential recovery practices and habits such as sleep, stress management, and physical activity, further exacerbating health inequities.

**Subtheme 6c: One Size Does Not Fit All.** Participants expressed frustration with the non-personalized, one-size-fits-all approach to stroke care, which they felt hindered their recovery, overall well-being, and recovery journey. This one-size-fits-all approach often failed to account for the unique needs of younger adult women who manage multiple responsibilities across their personal and professional responsibilities. Instead, participants expressed frequently being treated as an older adult with care plans, rehabilitation, and prescribed medications based on the standard stroke care that they do not fit. One participant shared, “I just feel like I got treated like an 80-year-old woman that had a stroke. I was put on cholesterol medication[*s*], but I didn't even need them, and my cholesterol dropped to dangerously low levels of cholesterol” (Participant 3). Similarly, another participant indicated:

In the hospital, there was a lot of comparison to averages. I don't know if there is enough consideration to how you were before the stroke versus after the stroke, right? So, we are individuals, not averages, and I got frustrated by that quite a bit. (Participant 9)

Participant 5 further explained:

Losing one’s license for, maybe, someone who's 80 or 90, I'm sure that is difficult, but I'll be frank, for a woman in their 40s, when they're responsible for taking kids to school, taking kids to different things, to lose their driver's license, that had serious impacts in terms of just losing your independence. You no longer have independence to be able to do things for yourself at all, and so the independence you lose. And, I think, the mental health impacts are crazy, too.

The one-size approach also centered around constantly being compared to the “average” with little regard to who they were pre-stroke.

## Discussion

This study offers critical insight into the lived experiences of younger adult women who had a stroke or are of high stroke risk, revealing how intersecting identities such as gender, age, social, and structural determinants of health shape lifestyle habits, access to care, health outcomes, and recovery. By applying an intersectionality lens, we can better understand and appreciate the complex interplay between individual experiences, systemic, and structural inequities and how these dynamics may pose as a facilitator or barrier for women’s stroke-related journey and recovery. The findings have the potential to address the paucity of research on gender, brain health, and stroke in order to bridge a much overdo gap in knowledge and science in women’s health.

### Perceived facilitators to brain health and recovery through an intersectional lens

**Fig. 2:**
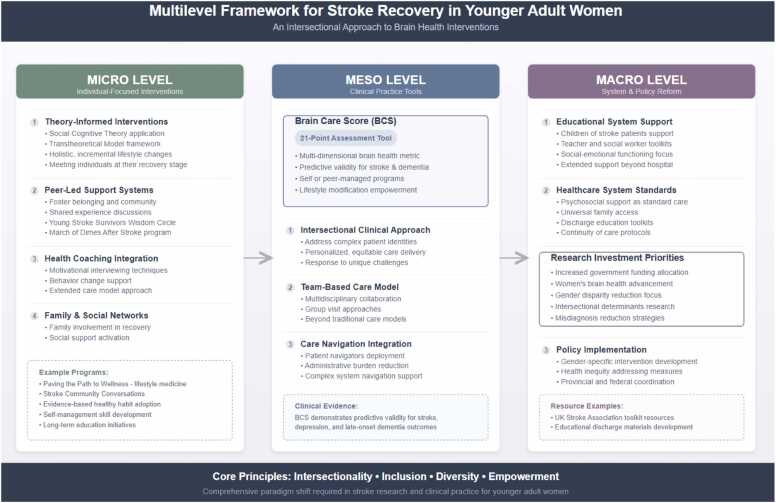
Overview of implications.

A spectrum of perceived facilitators were found that supported women’s brain health and lifestyle habits which are interconnected through the following: 1) *wellbeing care* embracing a holistic approach—mind, body and spirit—to recovery (e.g., having a positive attitude and different outlook on life and joining various art and exercise groups), 2) *lifestyle care* improving lifestyle habits (e.g., healthy eating, reducing alcohol consumption and stress levels, and increasing physical activity), 3) *social care* including social support such as partners helping more with child and household responsibilities, children adapting their behavior to ensure adequate time, healing, and space for their healing, and 4) *self-care*, i.e., giving themselves “permission” to prioritize themselves and recovery while adapting to social challenges (Fig. 2).

The personal strength and family support, which are in line with the self-determination theory (Ryan & Deci, 2000) and the theory of coping (Lazarus, 1974) were similarly identified as facilitators for the management of multiple chronic illnesses (Koch et al., 2015). Additionally, embracing a holistic approach and adopting healthy lifestyle habits has been shown to reduce stroke risk and improve overall secondary stroke prevention, e.g., reduce blood pressure, cholesterol levels, and improve engagement in physical activity, mental health, and social functions and connections (Bailey, 2018, Thomas et al., 2022).

These perceived facilitators are not uniformly experienced. Rather, they are shaped by the intersection of multiple social identities and structural factors that influence women’s lived experiences. While some facilitators arise from individual behaviors and choices (e.g., diet change and engaging in physical activity), they are also outcomes embedded in power and privilege. For instance, some participants’ potential to access and benefit from these supports were influenced by their positionality: physical ability, race, identity, being younger, fluency in English, education, income, housing, community support, and being of higher socioeconomic status. These intersecting privileges provided them with greater health literacy, communication and system navigation skills, and the financial means to support their recovery. At the same time, their experiences highlight the need for healthcare systems to recognize and respond to the diverse realities of women’s lives where their social and structural determinants of health can either facilitate or constrain their recovery (see also Berkhout et al., 2024a).

### Perceived barriers to women brain health and healthy lifestyle habits through an intersectionality lens

Women in this study described a spectrum of perceived barriers that impacted their brain health and lifestyle habits: (1) *well-being/mental health*, i.e., psychological impact of lived-experience (fear and anxiety of stroke recurrence), (2) *parental role*, with the negative impact of the stroke on children (particularly their social-emotional and psychological functioning) and on women (the overstimulation and overwhelming feeling experienced by women with mothering/parenting post-stroke), (3) *professional role* including grief and anxiety of abruptly leaving their careers and the pressures of returning to work, (4) *social role*, i.e., the gender socialization of women and their role in families, self-care, and overall health, and (5) *care experience* conditioned by the misdiagnosis, treatment, and administrative burden of the stroke on women within the healthcare system (Fig. 2).

From an intersectionality lens, the perceived barriers reflect the convergence of gender, social and structural determinants of health, (dis)ability, health status, access, language, and systemic bias on timely stroke and related diagnosis, care, and treatment. Even among the women with some privileges, they still encountered challenges with timely stroke-related diagnosis, care, and treatment. This is aligned with the literature. For instance, the risk of stroke misdiagnosis is nearly seven-fold among younger adults 18–45 vs. greater than 75 years (Newman-Toker et al., 2014). A potential explanation to this disparity is the knowledge gap surrounding atypical stroke symptoms in women (e.g., nonfocal symptoms such as fatigue, cognitive changes, headaches, and generalized weakness) (Berkhout et al., 2024b, Jerath et al., 2011, Rexrode et al., 2022).

Beyond the knowledge gap, women also experience the “trust gap” which is systemic bias where their experiences and symptoms are perceived as less credible. These experiences highlight how gendered assumptions and systemic biases within the current healthcare system and practices have the potential to negatively impact patient care and health outcomes. It is crucial to recognize that these barriers are not equally experienced among all women. Of note, for women whose social and structural determinants of health are compromised (e.g., non-English speaking, racialized, of lower socioeconomic status), these barriers are much more amplified, which may have more severe and negative health outcomes and long-term sequalae (Mital et al., 2021, Morgenstern et al., 2023).

The gender socialization of women emerged as a perceived barrier to women’s mental health, as highlighted not only in this study, but in the literature (see, e.g., Greenshield Cares, 2023). These findings reflect how gender socialization of women often result in prioritizing the needs of others over their own and making personal sacrifices in favor of their parental role and demands (Forbes et al., 2020, Moore and Abetz, 2019). Such prioritization often leads to diminished adoption and engagement in healthy behaviors (e.g., physical activity, healthy eating, and sleep) and lifestyle habits (McGannon and Schinke, 2013, Simpson et al., 2022) required support their own recovery.

Gender socialization is further compounded by the stressors of returning to work (RTW) post-stroke, as experienced by the women in the study. The stressors of RTW is common among younger adult patients who had a stroke and are influenced by many factors such as stroke sequalae (e.g., neurological deficits, cognitive ability, and fatigue), personal coping strategies, meaning of work, and preparatory environment (e.g., communication and cooperation from employers, accommodation and attitude of employer and organization, and uncertainty of stroke disclosure) (Brannigan et al., 2017, Edwards et al., 2018). In this study, while some women were able to RTW and had access to workplace accommodations and employment insurance, others did not have the same resources and supports. This disparity highlights the intersecting factors of employment type, education, and socioeconomic status on women’s abilities to access workplace-related resources post-stroke. Furthermore, it reflects broader systemic inequities where women with multiple marginalized identities such as being single mothers, underrepresented of lower socioeconomic status and with precarious employment experience more complexities and challenges with navigating their RTW post-stroke.

## Implications

Findings from this study have several implications to practice (Fig. 2) from a micro, meso, and macro level. At a micro level, it is recommended to have intensive, long-term education and behavioral theory-informed (e.g., social cognitive theory and the transtheoretical model) interventions that focus on holistic and incremental lifestyle changes that meet persons where they are in their recovery and that involve family and social support (Bailey, 2018). Second, having peer-led and group-based interventions has the potential to foster a sense of belonging, community, social connectedness that meets the unique and varying needs and goals of younger adult women. Further, having peer-led interventions would provide a sense of hope, the ability to discuss challenges among persons with similar experiences, and provide a sense of belonging (Ibrahim et al., 2025, Wijekoon et al., 2020). For instance, in Canada, there is a multi-platform March of Dimes after-stroke program that offers peer-support groups such as a stroke community conversation and young stroke survivors wisdom circle in addition to resources, toolkits, and supports to people who had a stroke (March of Dimes, 2025). Furthermore, it is recommended to utilize motivational interviewing and integrate health coaching within interventions to support behavior change (Askari et al., 2024) and secondary stroke prevention that extends beyond the current and traditional model of care (Bailey, 2018, Frates et al., 2017, Frost et al., 2018). Such programs are emerging. For example, the Paving the Path to Wellness program provides education on evidence-based topics related to lifestyle medicine with the goal of supporting the long-term adoption of healthy habits (Comander et al., 2021).

At a meso-level, it is recommended to use practical tools such as the Brain Care Score (BCS) in clinical practice and particularly in the primary care setting. The BCS is a 21-point multi-dimensional and motivational tool used in clinical practice to empower and guide for modifying lifestyle habits to improve brain health and follows over time with self-managing or peer- vs. provider-managing programs (Singh et al., 2022). The BCS can be widely used as a proactive approach in the primary care setting to support primary and/or secondary stroke prevention and has demonstrated clinical relevance and predictive validity for stroke, depression, and late-onset dementia (Singh et al., 2023, Singh et al., 2024). When applied through an intersectional lens and in the primary care setting, tools like the BCS can help primary care providers to better understand and respond to the unique challenges and complex identities and needs of patients, ensuring more equitable and personalized approach to patient care.

At a macro-level, it is important to ensure children are afforded the necessary support and services to facilitate their coping. Support and services ought to be provided in the healthcare system (e.g., throughout the mother’s hospital stay and via educational toolkits during discharge) and extended into the school system. As such, equipping teachers and social workers with the necessary toolkits and resources (UK Stroke Association, 2021) is integral to supporting children’s social-emotional and psychological functioning as part of the post-stroke recovery and journey.

From a clinical perspective, there is a need for better integration of case management and patient navigators in the current stroke model of care to minimize the healthcare administrative burden on patients to support them with navigating the complex healthcare system in their recovery and journey (Giardino & De Jesus, 2023).

Furthermore, appreciating that stroke is not an isolated incident, it is important for the current stroke model of care to ensure appropriate and psychosocial support (Winstein et al., 2016) and that initiatives are “standard of care” available to all families from an intersectional lens and approach.

From a research perspective, there is also an urgent need for increased investment from provincial and federal governments to advance women’s brain health, which continues to be underfunded despite its growing relevance and impact (Koirala et al., 2023). The lack of funding perpetuates gender-based disparities in research and care, particularly for women facing intersecting social and structural determinants of health-related challenges.

## Limitations

This study had some limitations. First, the findings are based on a study conducted in a single academic health sciences center in Toronto, Canada, which may impact the transferability of the findings to other settings and contexts. Second, although efforts were employed to recruit women of diverse cultural and ethnic backgrounds, most of the study sample were women who identified as white, Canadian born, speaking English as their first language, and overall, of higher socioeconomic status (SES). As such, women of diverse cultural backgrounds and ethnicities and with varying SES levels may have different experiences, facilitators, and barriers to the study participants. Further efforts are being made by the research team to develop partnerships with organizations and community centers that serve socially and structurally marginalized populations to address this limitation for future initiatives and studies. Finally, while the research team attained data saturation, there is a potential that not all information related to the participant’s perspectives was communicated during the focus groups.

## Conclusion

Understanding the needs around brain health care, particularly from an intersectionality lens, is central in women’s health and highlights the importance for a paradigm shift in stroke research and practice. A total of six themes emerged that offer insight into the lifestyle-related knowledge, practices, and associated facilitators and barriers specific to women who had a stroke or are at high risk of stroke. This is coupled with recommendations on the design and development of brain health and stroke-related interventions to support recovery, overall health, wellbeing, and adoption of healthy lifestyle habits. Efforts to optimize brain health from an intersectional approach should be made for everyone, particularly women as the most impacted by stroke and neurological conditions, respecting the principles of inclusion, diversity, and empowerment.

## Ethics Statement

The studies involving human participants were reviewed and approved by the institutional Quality Improvement Committee at the University Health Network. The study was conducted in accordance with the study protocol, institutional requirements, and the Declaration of Helsinki.

## Author Contributions

SI, EK led the data analysis and preparation of the manuscript. SI and LZ participated in the data collection. AP secured the funding and supervised the study. VR led and advised on all components of the study methods, and interpretation of study findings. All authors contributed to the overall study design, reviewed, and approved of the manuscript.

## Author Contributions

SI and EK led the data analysis and preparation of the manuscript. SN and LZ contributed to participant recruitment process, reviewed and approved the manuscript. VR advised on the study methods, interpretation of study findings, and reviewed and approved the manuscript. AP secured the funding, supervised the study conception and design, data collection, interpretation of the study findings, manuscript preparation, and reviewed and approved of the manuscript. All authors contributed to the overall study design as well as reviewed and approved of the manuscript. The authors did not use any artificial intelligence (AI)-assisted technologies in the creation of the submitted work.

## Funding

This work was supported by funding from the University of Toronto Division of Neurology (The Slamen-Fast New Initiatives in Neurology).

## CRediT authorship contribution statement

**Jasper R. Senff:** Writing – review & editing, Writing – original draft. **Jonathan Rosand:** Writing – review & editing, Writing – original draft. **Sarah Ibrahim:** Writing – review & editing, Writing – original draft, Visualization, Methodology, Formal analysis. **Sanjula D. Singh:** Writing – review & editing, Writing – original draft. **Emine Kocabas:** Writing – review & editing, Writing – original draft, Formal analysis. **Valeria E. Rac:** Writing – review & editing, Writing – original draft, Methodology, Investigation. **Lindsey Zhang:** Writing – review & editing, Project administration, Data curation. **Aleksandra Pikula:** Writing – review & editing, Writing – original draft, Supervision, Investigation, Funding acquisition, Data curation, Conceptualization. **Angela Verven:** Writing – review & editing, Writing – original draft, Visualization. **Syeda Hashmi:** Writing – review & editing. **Sharon Ng:** Writing – review & editing, Writing – original draft, Data curation. **Troy Francis:** Writing – review & editing, Writing – original draft. **Aleksandra Stanimirovic:** Writing – review & editing, Writing – original draft. **Judith Coulson:** Writing – review & editing.

## Declaration of Competing Interest

The authors declare the following financial interests or personal relationships which may be considered as potential competing interests: Dr. Pikula receives support from the Jay and Sari Sonshine Chair in Stroke Prevention and Cerebrovascular Disease at University of Toronto/University Health Network. The senior author receives support from the Jay and Sari Sonshine Chair in Stroke Prevention and Cerebrovascular Disease at University of Toronto/University Health Network. Other authors declare that they have no known competing financial interests or personal relationships that could have appeared to influence the work reported in this paper.

## Data Availability

The original contributions presented in the study are included in the article. Further inquiries can be directed to the corresponding author.
